# Hydatid Disease of the Femur with an Extraosseous Extent due to a Former Biopsy Complicated by a Pathological Fracture

**DOI:** 10.1155/2012/169545

**Published:** 2012-07-30

**Authors:** M. Ciftdemir, A. Sezer, F. O. Puyan, C. Copuroglu, M. Ozcan

**Affiliations:** ^1^Department of Orthopaedics and Traumatology, Faculty of Medicine, Trakya University, 22030 Edirne, Turkey; ^2^Department of General Surgery, Faculty of Medicine, Trakya University, 22030 Edirne, Turkey; ^3^Department of Pathology, Faculty of Medicine, Trakya University, 22030 Edirne, Turkey

## Abstract

Hydatid disease of the bone represents about 1–2.5% of all human hydatid disease. Spine is the most affected part of the skeleton with 50% incidence of all bone hydatidosis. Extraspinal bone hydatidosis is much rare. Diagnosis is difficult in the bone hydatid disease. Bone tumors, tumor-like lesions, and specific and nonspecific infections should be considered in the differential diagnosis. Radiological, laboratory, and clinical findings combined with strong element of suspicion are the key for diagnosis. Bone biopsies should be avoided because of the danger of anaphylaxis, sensitization, and spread. This paper describes the management of a patient with primary hydatidosis of the femur, which had been complicated by an extraosseous involvement, cortical erosion, and a pathological fracture due to a former needle biopsy.

## 1. Introduction

Hydatid disease caused by *Echinococcus granulosus* is a zoonosis which human beings occasionally become intermediate hosts. Hydatid cysts may develop anywhere in the body. It is mostly found in liver and lungs. The incidence of bone hydatidosis is about 1–2.5% of all human hydatid disease [1]. Spine is the most affected part of the skeleton with 50% incidence of all bone hydatidosis among humans.

Primary bone hydatidosis develops when the scoleces are localized in the bone. Cysts within the bone may remain asymptomatic for years. Osseous tissue limits the expansion of hydatid cyst. In bone hydatidosis, daughter vesicles develop rather than a single cyst because of the dense and hard structure of the bone. Cysts spread slowly in medullary cavity eroding the bony trabeculae. Patients may complain of pain but often delay consulting a physician until neural compromise or pathologic fracture.

This paper describes the management of a patient with primary hydatidosis of the femur, which had been complicated by an extraosseous involvement, cortical erosion, and a pathological fracture due to a former needle biopsy.

## 2. Case Report

**Figure 1:**
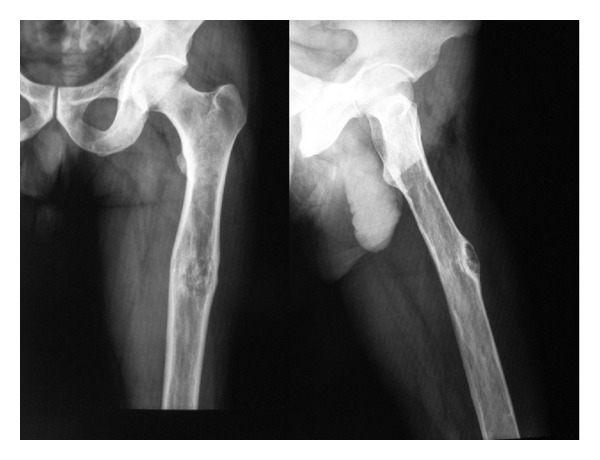
Plain radiographs of the left femur demonstrating moderate expansion in bone, including lytic areas with a cortical defect at the anterolateral aspect of the upper third of the left femoral shaft and cortical thinning.

A 50-year-old man, labourer in textile industry, has been admitted to our clinic with pain and swelling in his left thigh. The patient had a needle biopsy 6 years ago in another hospital because of a suspicious lesion seen on plain radiographs. Histopathological result revealed a hydatid cyst of left femur. Surgical treatment was recommended at that time, but the patient did not accept the recommended surgery. The patient has been treated with albendazole and mebendazole in irregular and interchanging episodes for 6 years. In physical examination, a large, immobile, and painless mass found at the anterolateral surface of the left thigh and lumping due to mild pain was noted. Laboratory studies revealed normal results; erythrocyte sedimentation rate was 12 mm/h (8–12 mm/h); haemoglobin and leucocyte counts were 14.9 g/dL (12.7–17.2 g/dL) and 9800/mL (4600–10200/mL), respectively, with an eosinophil percentage of 3.2% (0.5–7%). Indirect hemagglutination test (IHA) for *Echinococcus* was negative. Plain radiographs demonstrated moderate expansion and cortical thinning in bone, with a cortical defect at the anterolateral aspect of the upper third of the left femoral shaft (Figure 1). No signs of echinococcal disease were found in abdominal ultrasonography and computerized tomography (CT) of thorax.

**Figure 2:**
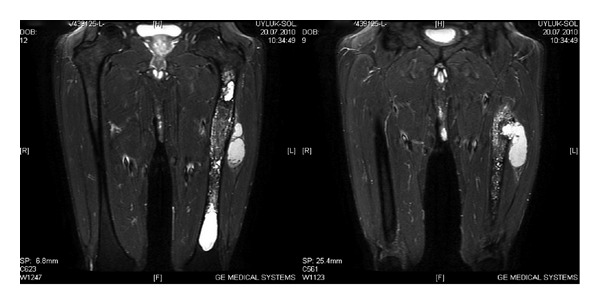
T2-weighted MRI views showing the bony involvement and the soft tissue extent.

Magnetic resonance imaging (MRI) revealed a lesion filling the whole intramedullary cavity of the left femur from intertrochanteric region to the distal metaphysis with heterogeneous signal features. Besides, a 6 × 3 cm cystic lesion destructing the lateral femoral cortex at the proximal diaphyseal region, expanding anteriorly into vastus lateralis and vastus intermedius muscles, was also noted in MRI (Figure 2).

Surgical debridement took place after 5 days of oral treatment with albendazole 10 mg/kg/day. At the surgery, extraosseous portion of the cyst exiting from the cortical defect at the lateral cortex has been dissected from a lateral femoral approach and excised 10 minutes after injection of 50 mL 10% povidone-iodine solution into the cyst. Osseous defect at the lateral femoral cortex has been closed in a waterproof fashion with bone wax and surrounded with povidone-iodine-soaked gauze. Left knee joint was opened via a medial parapatellar approach. Intercondylar notch has been drilled to the distal femoral metaphysis. An 8 mm endotracheal intubation tube has been inserted into the intercondylar notch, and its cuff has been inflated to prevent any leakage. Using a direct lateral approach, proximal entry point for proximal femur has been formed from the fossa piriformis. Povidone-iodine solution has been injected through the intubation tube into the medullary cavity. After 10 minutes, all daughter vesicles and germinative membranes aspirated safely from the distal end through the intubation tube, which has been placed previously, while aspirating simultaneous drilling and broaching the medullary cavity of the left femur from proximal to distal has been performed. The defect at the lateral femoral cortex has been preserved for any leakage during this process. When broaching has been finished, the medullary cavity has been exposed to 70% of phenol solution for 5 minutes. After irrigation with saline solution, the wound has been closed in layers over suction drains settled in the medullary cavity. No anaphylactic reaction has been observed during the surgery.

**Figure 3:**
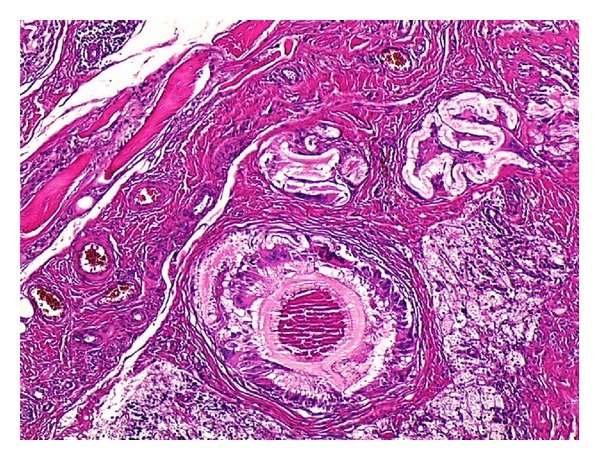
Lamellar cuticular membrane with giant cell and histiocytic reaction seen between the fibrovascular tissues in pathological specimen.

Oral albendazole 10 mg/kg/day was prescribed at the postoperative period for two months. Drains have been removed at the postoperative third day. Pathological analysis of the specimens was reported as echinococcal hydatid cysts of femur (Figure 3). The patient has been discharged nonweight bearing with crutches.

**Figure 4:**
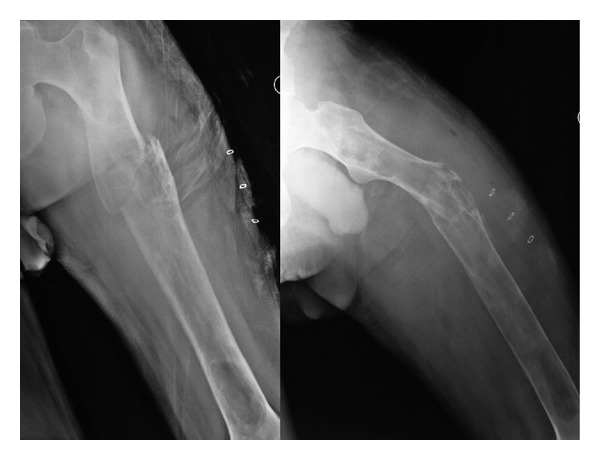
Pathological fracture of the left femur at the level of cortical destruction.

**Figure 5:**
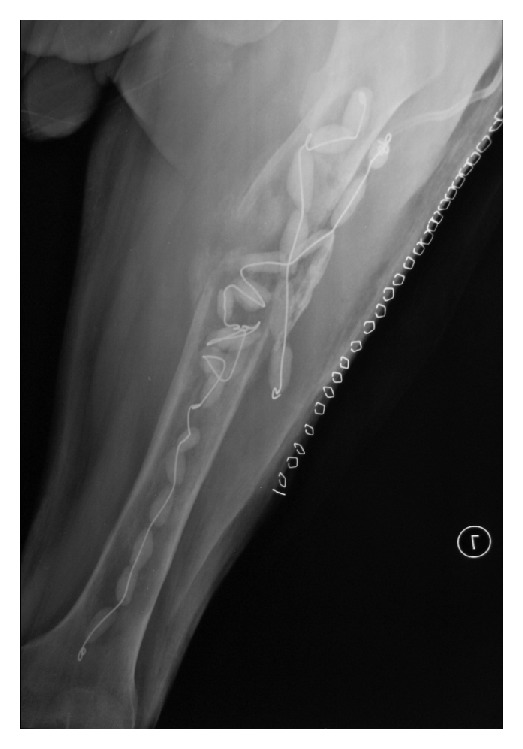
Medullary cavity of the femur filled with custom-made vancomycin beads.

**Figure 6:**
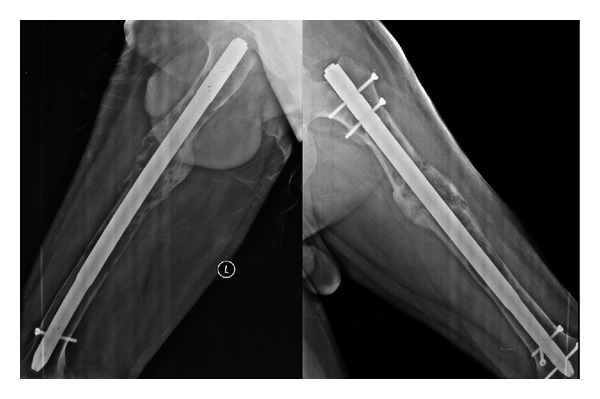
Pathological fracture treated with custom-made interlocking intramedullary nail. Union is seen at the sixth month postoperatively.

Ten days after the discharge, the patient came back with a spontaneous pathological fracture of the left femur (Figure 4). A haemorrhagic drainage was also present from the wound. A thorough debridement took place immediately. No evidence of hydatid disease has been observed intraoperatively. Biopsies were taken from muscle layers and bone for microbiology. Results of the microbiological cultures revealed a methicillin-sensitive *Staphylococcus aureus* infection. Also no scolex was seen at the specimens. In intramedullary cavity filled with vancomycin beads, wound has been closed, and the patient has been put into skeletal traction from tuberositas tibia (Figure 5). Infection has been healed after multiple debridements and 6 weeks of parenteral cefazolin sodium 3 gr/day and rifampicin 500 gr/day treatment combined with oral albendazole. When the infection has ceased, the pathological fracture has been treated using a custom-made interlocking intramedullary nail (Figure 6). No early or late postoperative complications were seen. The patient was discharged at postoperative tenth day walking partial weight bearing with a pair of crutches. At sixth month, the patient was able to walk full weight bearing with radiological signs of bony union.

## 3. Discussion

Bone hydatidosis is a rare condition especially seen in endemic areas. Spine is the most common location (50%); extraspinal bone hydatidosis is much rare. This describes the management of a patient with primary hydatidosis of the femur, which had been complicated by an extraosseous involvement, cortical erosion, and a pathological fracture due to a former needle biopsy.

The diagnosis of bone hydatidosis is difficult and is easily overlooked unless there is a strong element of suspicion [1]. Tumors and tumor-like lesions of the bone as well as tuberculosis and some fungal infections should be considered in the differential diagnosis. Bone hydatidosis is often asymptomatic for a long period and is usually detected only after a pathologic fracture, secondary infection, or neurovascular symptoms caused by compression [2]. Casoni intradermal test and Weinberg complement fixation test were former diagnostic tools, which have been disused due to false positive results and lesser sensitivity in extrahepatic hydatidosis [3]. Preoperative definitive diagnosis is often impossible in bone hydatidosis. Immunological tests are usually negative in extrahepatic disease. Plain radiographic findings of bony hydatidosis are nonspecific. CT has shown to be the best imaging tool for diagnosis and preoperative planning in bone hydatidosis [4]. MRI is helpful in evaluating the soft tissue and bone marrow involvement. Fine-needle aspiration must be avoided because of the danger of spread, sensitization, and anaphylaxis [1, 5, 6]. The case described above had undergone needle biopsy 6 years ago due to a suspicious lesion in left femoral shaft. The hydatid cyst continued to grow and has bulged outside from the cortical defect, which has been created by the biopsy needle.

Bone hydatid disease should be considered as a progressive tumor to get successful results [7]. Safe excision with wide margins should be addressed in the surgical treatment of bone hydatidosis. Local chemical and physical adjuvants such as formalin, silver nitrate 0.5%, phenol 70%, and local heat are often used to prevent recurrence. The forming defect after curettage or excision is a major problem. The dead space can be filled with bone grafts, bone cement, or endoprostheses. Bone grafts can be invaded by the parasites, and recurrence is often [8]. Yildiz et al. have reported 10 patients having bone hydatidosis treated with curettage, swabbing with povidone iodine and filling the defect with polymethylmethacrylate (PMMA) bone cement. Three of the 7 patients who have been followed up for more than 5 years had recurrences [9]. The authors state that curettage and PMMA application may not eradicate bone hydatidosis, but lower the rate of recurrence. Tomak et al. have reported a patient with hydatidosis of the femur treated with curettage and filling the defect with PMMA [7]. The patient was disease-free after 6 years. PMMA may be considered as an appropriate material to fill such defects because of heat production and toxic polymerisation, but PMMA has negative effects on fracture healing. Herrera and Martinez have reported 26 extraspinal bone hydatidosis cases. Two of the patients with hydatidosis of femur have been treated with curettage and external fixation to reinforce the femoral shaft until the lesion healed. The external fixators were left in place for 5 months and 7 months in 2 patients [1].

## 4. Conclusions

Hydatid disease filling the entire medullary cavity of the femur with a cortical defect and an extraosseous extent created a challenging condition in our case. A stepped surgical care has been planned for the patient. Our plan was to excise the extraosseous portion initially and then to decompress the medullary cavity of the femur safely in the first step. Filling the entire medullary cavity with PMMA has not been considered for this particular patient. Reinforcement of the femoral shaft using an intramedullary nail has been considered for the next step because of the possibility of postoperative infection and remnant cysts. Prognosis of the patient has been worsened because of the pathological fracture and infection at the early postoperative period. The infection has been healed after 6 weeks of antibiotics and consecutive bone and soft tissue debridements. Finally, the fracture has been treated using a custom-made intramedullary nail that fits into the widened medullary cavity after the infection has been healed.
